# Predicting the Environmental Suitability and Identifying Climate and Sociodemographic Correlates of Guinea Worm (*Dracunculus medinensis*) in Chad

**DOI:** 10.4269/ajtmh.23-0681

**Published:** 2024-07-09

**Authors:** Obiora A. Eneanya, Maryann G. Delea, Jorge Cano, Philip Ouakou Tchindebet, Robert L. Richards, Yujing Zhao, Abdalla Meftuh, Karmen Unterwegner, Sarah Anne J. Guagliardo, Donald R. Hopkins, Adam Weiss

**Affiliations:** ^1^ Guinea Worm Eradication Program, The Carter Center, Atlanta, Georgia;; ^2^ Expanded Special Project for Elimination of Neglected Tropical Diseases, World Health Organization, Brazzaville, Republic of Congo;; ^3^ Guinea Worm Eradication Program, Ministry of Public Health, N’Djamena, Chad;; ^4^ School of Biological Sciences, Georgia Institute of Technology, Atlanta, Georgia;; ^5^ Guinea Worm Eradication Program, The Carter Center, N’Djamena, Chad;; ^6^ Parasitic Diseases Branch, Division of Parasitic Diseases and Malaria, Centers for Disease Control and Prevention, Atlanta, Georgia

## Abstract

A comprehensive understanding of the spatial distribution and correlates of infection are key for the planning of disease control programs and assessing the feasibility of elimination and/or eradication. In this work, we used species distribution modeling to predict the environmental suitability of the Guinea worm (*Dracunculus medinensis*) and identify important climatic and sociodemographic risk factors. Using Guinea worm surveillance data collected by the Chad Guinea Worm Eradication Program (CGWEP) from 2010 to 2022 in combination with remotely sensed climate and sociodemographic correlates of infection within an ensemble machine learning framework, we mapped the environmental suitability of Guinea worm infection in Chad. The same analytical framework was also used to ascertain the contribution and influence of the identified climatic risk factors. Spatial distribution maps showed predominant clustering around the southern regions and along the Chari River. We also identified areas predicted to be environmentally suitable for infection. Of note are districts near the western border with Cameroon and southeastern border with Central African Republic. Key environmental correlates of infection as identified by the model were proximity to permanent rivers and inland lakes, farmlands, land surface temperature, and precipitation. This work provides a comprehensive model of the spatial distribution of Guinea worm infections in Chad 2010–2022 and sheds light on potential environmental correlates of infection. As the CGWEP moves toward elimination, the methods and results in this study will inform surveillance activities and help optimize the allocation of intervention resources.

## INTRODUCTION

Dracunculiasis is one of the diseases targeted for eradication by the World Health Organization (WHO).^1^ The global reduction in overall Guinea worm case numbers represents one of the most remarkable public health achievements in history. Soon after the inception of the Guinea Worm Eradication Program in the 1980s, there were an estimated 3.5 million human cases^2^; in 2022, just 13 human cases were reported globally^3^ (six in Chad, five in South Sudan, one in Ethiopia, and one in Central African Republic).^4^ Despite the achievements made toward eradication of Guinea worm, several challenges remain, including 1) the detection of canine cases in Chad in 2012,^5^ 2) the suggested involvement of paratenic and/or transport hosts such as fish, frogs, or other aquatic animals in the transmission cycle,^6^^,^^7^ 3) insecurity, conflict, and civil unrest in endemic areas, which have inhibited the efficient deployment of surveillance and intervention measures,^8^ and 4) detection of cases and infections in hard-to-reach and/or border villages, risking exportation to new geographies.^3^

Chad remains the country with the highest annual number of reported Guinea worm infections in both humans and animals (mostly in domestic dogs and cats) in recent years.^3^ The number of infections detected in domestic dogs in Chad increased from 27 in 2012 to 1,927 in 2019, although as of the end of 2022, only 456 infected domestic dogs were detected. Simultaneously, the number of villages under active surveillance increased from 342 in 2012 to more than 2,000 in 2022. The number of reported Guinea worm infections raises questions about whether there are additional areas within the country, besides the existing well-known endemic areas, where environmental conditions would support *Dracunculus medinensis* transmission and which are not currently under active surveillance.

Guinea worm surveillance infrastructure in extant endemic countries involves both active surveillance and passive surveillance. A community-based approach is taken when implementing active surveillance, which involves community members systematically searching for Guinea worm disease or signs and symptoms thereof in human and animal hosts. Although the scale of the Guinea worm surveillance infrastructure in Chad is impressive, the initiation of active surveillance in any given area has historically been reactive in nature. This is because villages are only placed under active surveillance when 1) a new Guinea worm case or animal infection is detected, 2) the villages are near other villages where Guinea worms have been detected, or 3) the villages share common epidemiological risk factors such as shared water sources, fishing sites, farmlands, or familial relationships with other villages where Guinea worms have been detected. In contrast, passive surveillance does not involve systematic active searches; instead, it relies on reports of suspected or rumored Guinea worm infections that are conveyed through the local health system, such as health centers within the villages or national reporting hotlines. It is important to note that dracunculiasis is a reportable disease. Furthermore, previously endemic villages remain under active surveillance after they no longer report cases. Transitioning a village to passive surveillance is done at the discretion of the national program based on current epidemiology in the zone and would follow at least 3 consecutive years without any reported cases in that village or those adjacent. Therefore, predictive geospatial models enable proactive identification of potential transmission hotspots and inform decisions about which areas to place under active and passive surveillance. In addition, geospatial models can be used to characterize spatially explicit habitat suitability by associating species distribution data with potential disease correlates such as climate and environmental and sociodemographic factors. This approach has been extensively used to describe the geographical extents and environmental correlates of several other neglected tropical diseases (NTDs) such as human African trypanosomiasis, lymphatic filariasis, schistosomiasis, onchocerciasis, and trachoma.^9^^–^^11^ An understanding of the possible geographical distribution of the Guinea worm using high-resolution risk maps is key for surveillance, control, and eradication efforts.

In this work, using Guinea worm surveillance data collected by the Chad Guinea Worm Eradication Program (CGWEP) from 2010 to 2022, we modeled the environmental suitability for the occurrence of Guinea worm infections in Chad. This model was then used to map the distribution of infection, delineating areas suitable for disease transmission, and identify climate, environmental, and sociodemographic correlates of Guinea worm infection.

## MATERIALS AND METHODS

### Survey area and ecological zones.

Chad is divided into four main ecological zones^12^; the drier northern region is predominantly Sahelian with mountainous areas along the northern border with Libya, whereas the wetter, greener, and more populous southern region is either Sudanian or tropical. N’Djamena, which is the capital, is characterized as urban.

### Guinea worm surveillance data.

**Figure 1.**
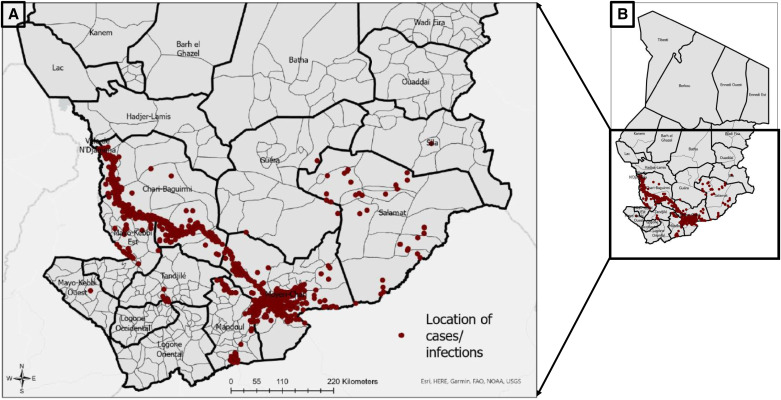
Location of villages reporting Guinea worm infection in all hosts from 2010 to 2022. (**A**) Subset of Chad highlighting locations of Guinea worm endemic villages. (**B**) Insert is the entire map of Chad, indicating Guinea worm endemic villages.

Guinea worm infections have been predominantly detected along the Chari River basin in Chad, which runs from Lake Chad near the western border with Cameroon to the southeastern border with Central African Republic. Surveillance data collected by the CGWEP 2010–2022 was used for this analysis (Figure 1).

Surveillance infrastructure for the CGWEP has been described in detail elsewhere.^5^ Briefly, community-based active surveillance is conducted to identify Guinea worm infections. For villages under active surveillance, village volunteers conduct daily searches for Guinea worm, and when a suspected Guinea worm infection or case is identified, a CGWEP supervisor is sent to verify. If signs/symptoms are consistent with Guinea worm, worms are extracted, and relevant programmatic and epidemiological investigations are carried out. Worm specimens from humans are then transported to the CGWEP reference laboratory (U.S. Centers for Disease Control and Prevention, Atlanta, GA) for confirmation. Not all specimens from animals are sent to the reference laboratory because of the large volume of such specimens, though they are received by other laboratories for various analyses. Worm specimens from animals may be transported to the reference laboratory if the CGWEP supervisors in the field are unable to verify the diagnosis in the field and/or if the specimens have epidemiological significance. Owing to the small number of human cases remaining, surveillance efforts mostly identify infections in domestic dogs and cats. Studies of the genetic variations of Guinea worm DNA from several hosts suggest that the same Guinea worm parasite populations infect various definitive host species (i.e., genetically similar *D. medinensis* worms infect both humans and animals).^13^ Therefore, in this analysis, any villages that had reported a case of Guinea worm between 2010 and 2022 were considered “positive,” regardless of host. This analysis was implemented using data from 6,489 village-level infection data points (6,198 dog worms, 150 cat worms, and 141 human worms).

### Ethical approval.

The process of obtaining ethical approvals, obtaining informed consent, and arranging logistical procedures for field surveys was handled in-country by the CGWEP, with technical support provided by The Carter Center. Analysis of Guinea worm surveillance data was previously reviewed by the U.S. CDC and was determined to be nonresearch; it was conducted consistent with applicable federal law and CDC policy (e.g., see 45 C.F.R. part 46, 21 C.F.R. part 56; 42 U.S.C. §241(d); 5 U.S.C. §552a; 44 U.S.C. §3501 et seq.)

### Climate, environmental, and sociodemographic data.

In total, we used 15 covariates for this analysis (Table 1). All input grid raster covariates were resampled to a common spatial resolution of 1 × 1 km^2^ using the nearest-neighbor algorithm^24^ and then clipped to align to the geographical extents of Chad. Raster manipulation and processing were done using the *raster* package in R.^25^ Values that corresponded to the location of infection data points were extracted from the stack of raster covariates.

**Table 1:** Climate, environmental, and sociodemographic covariates that were integrated into the models and their sources

Variables	Source
Land Surface Temperature (°C)	WorldClim^14^
Precipitation (mm)	
Population Density (%)	WorldPop^15^
Elevation (m)	
Slope of Terrain (°)	
Night Light Emissivity (W·sr^−1^·m^−2^)	
Euclidean Distance to Permanent Water Bodies (m)	
Euclidean Distance to Temporary Water Bodies (m)	
Euclidean Distance to Edge of Nature Reserves (m)	
Croplands/Farmlands (%)	The Global Croplands Dataset^16^
Vegetation Cover (%)	European Space Agency’s Globcover Project^17^
Livestock (cattle) Distribution (%)	Gridded Livestock of the World (GLW3) Database^18^^,^^19^
Food Insecurity Hotspots (gridded scale ranging from famine to food secure)	Famine and Early Warning Systems Network^20^^,^^21^
Ground/Terrestrial Water (mm)	NASAs Gravity Recovery and Climate Experiment (GRACE) Project^22^
Conflict Events (No. of fatalities resulting from conflict events)	The Armed Conflict Location & Event Data (ACLED) Project^23^

All covariates used in this analysis were either hypothesized or are known to be associated with Guinea worm transmission. The use of remotely sensed climate, environmental, and sociodemographic data in prediction has greatly increased in recent years. These data are generated either by orbital satellites or by extrapolation from point-level data collected through weather stations and nationally representative surveys. Incorporating covariates with known or hypothesized biological links to disease transmission into geospatial modeling improves model predictions.^26^

We downloaded climate data related to temperature and precipitation from the WorldClim v. 2.1 database,^14^ which provides interpolated long-term averages of climate and global weather data obtained from ∼15,000 weather stations distributed across the world. Also, data on population density, elevation, slope of terrain, nighttime light emissivity, and Euclidean distance to surface water bodies and the edge of nature reserves were all downloaded from the WorldPop repository.^15^

Because farm-related owner occupations are a correlate of Guinea worm infections in dogs,^27^ we considered covariates that were designated as farming communities or croplands. The Global Croplands dataset represents the proportion of land areas used for agricultural cultivation.^16^ Satellite data from Moderate Resolution Imaging Spectroradiometer and Satellite Pour I’Observation de la Terre Image vegetation sensors were combined with agricultural inventory data to build these global datasets of croplands. Also, vegetation cover types were extracted from the GlobCover project at the European Space Agency.^17^ Maps were derived by an automatic and regionally tuned classification of 300-m resolution imaging spectrometer sensor on the ENVISAT satellite mission. Vegetation cover types were in keeping with the United Nations land cover classification system.^28^ We considered livestock distribution in our analysis, specifically, cattle distribution. These data were downloaded from the Gridded Livestock of the World database.^18^^,^^29^

Furthermore, covariates for freshwater availability and food insecurity hotspots were also considered. Here, the availability of freshwater was defined as terrestrial or groundwater storage as observed by the Gravity Recovery and Climate Experiment satellites.^22^ This platform detects freshwater of different kinds, such as groundwater, soil moisture, surface waters, and wet biomass. In addition, the food insecurity hotspots dataset was downloaded from the Center for International Earth Science Information Network, Columbia University (input data were originally processed from the database of the Famine and Early Warning Systems Network).^20^^,^^21^

Finally, we generated a variable to account for conflict events and insecurity. Previous work suggests that conflict and civil unrest may potentially derail the program to eradicate the Guinea worm and other NTDs.^8^^,^^30^ For this variable, we extracted data from the Armed Conflict Location and Event Data (ACLED) project.^23^ Using the geostatistical technique, ordinary kriging,^31^ we built interpolated layers from the vector data of total number of fatalities due to civil unrest, ethnic clashes, and activities of extremist groups as recorded in the ACLED project. Hence, we created a raster layer with values across our geographical area of interest (i.e., Chad).

### Ensemble modeling.

Using village-level worm burden records (i.e., number of worms reported per village across species), we computed a variogram analysis to assess the spatial correlation in the reported Guinea worm infection data. Variograms measure the variability between pairs of data points^32^ and are often used to determine the distance at which clustering of infections occurs as well as the overall range across the geographical area of interest. Also, the existence of multicollinearity in the suite of covariates was explored by calculating variance inflation factors (VIFs), which represent the amount of variability that is explained by other covariates.^33^ Multicollinearity occurs when two or more covariates are not statistically independent and often leads to unstable estimates when used in statistical models.^33^ All covariates were retained for further analysis, as all had a VIF score of <10.

The detection (“presence”) of Guinea worm infection was considered for any village that reported at least one worm in any host. To build ecological niche models that require both presence and absence data, background (or pseudo-absence) data points are usually generated. Although true absence data points are the most ideal for building models, studies have shown that background data points are a suitable substitute.^34^ A random selection of 1,000 background data points was generated for this analysis. True presence and background data points were assigned similar weights.

To ensure that the best performing model was selected, we then implemented six different model algorithms: artificial neural networks, generalized boosted models (GBMs), generalized linear models, multivariate additive regression splines, random forest (RF), and surface range envelope. These algorithms are included within the Biodiversity Modeling (BIOMOD)^35^ framework and were implemented using the *biomod2* package in R (v. 4.2.1).^25^ The BIOMOD provides a robust computational framework for use in species distribution and allows for comparing different modeling algorithms using similar sets of dependent and independent data points.

To evaluate model accuracy, the models were trained using a random subset of 70% of the observed data, whereas the held-out 30% was used to test for prediction accuracy. We fitted the same set of training and evaluation data to all six model algorithms. The *biomod2* package in R offers functionality that allows for internal cross-validation. Here, a set number of data-splitting runs were computed whereby the model was fitted to one subset of the dataset and tested on the held-out subset. This internal cross-validation does not provide a measure of predictive performance per se, but provides a measure of internal consistency of models.^36^ We performed 100 model runs for each of the six algorithms, stored the evaluation values for each run, and then took the average for the final evaluation records. Here, evaluation was calculated as the area under the receiver operating characteristic (ROC) curve and the Hanssen-Kuipers discriminant (also known as the true skill statistic [TSS]). True skill statistic scores are often used in ecology as a metric for comparing the number of correct predictions minus predictions attributable to random guessing,^37^ while accounting for both sensitivity and specificity. A TSS score of +1 indicates a perfect score, 0 indicates random performance, and 0.5 or higher is generally considered an acceptable score for model performance.

The best-performing model algorithms (based on ROC and TSS scores) were then selected for final projection. Variable importance was determined by computing the percentage increment in mean square error by variable permutation. In addition, marginal effects plots were generated to explore the relationship between the suite of predictors used in the model and observed Guinea worm surveillance data. Finally, the predicted mean values of the probability of environmental suitability as well as uncertainty estimates were projected at a spatial resolution of 1 km × 1 km and clipped to the geographical extents of Chad. Maps were also presented as district-level aggregated mean values.

## RESULTS

### Variogram analysis.

**Figure 2.**
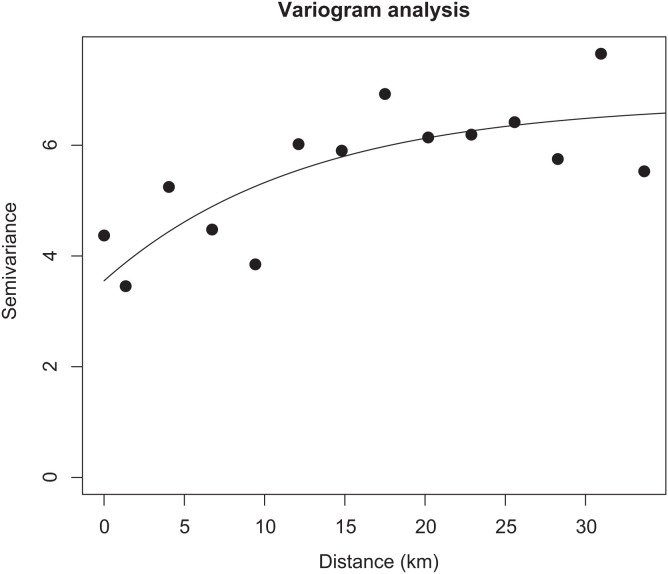
Variogram plot showing spatial correlation of the Guinea worm surveillance data. The empirical variogram is represented by the black dots; the theoretical variogram is represented by the black solid line.

The results from the variogram analysis (Figure 2) indicate that there is significant spatial correlation in the observed Guinea worm infection data (empirical variogram as represented by the black dots). The range of spatial correlation is approximately 35 km, after which spatial clustering starts to decay. The theoretical variogram (represented by the solid black line) is representative of a perfect scenario; the surveillance data used in this work generally align with the curve of the theoretical variogram.

### Model performance comparison.

**Figure 3.**
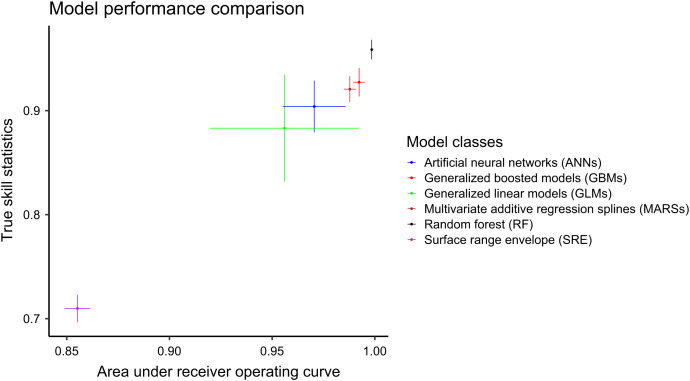
Model performance comparison as measured by area under the receiver operating characteristic and true skill statistic values of all model algorithms. The points represent the mean estimates, and the solid lines represents the 95% CIs.

The performance of the six model algorithms implemented using the BIOMOD package are shown in Figure 3. Although all the model algorithms performed reasonably well as measured by their ROC and TSS values, the RF model and GBM outperformed all model algorithms. Therefore, predictions from these two were chosen for constructing the final ensemble model.

### Identifying climate, environmental, and sociodemographic correlates of Guinea worm infection in Chad.

**Figure 4.**
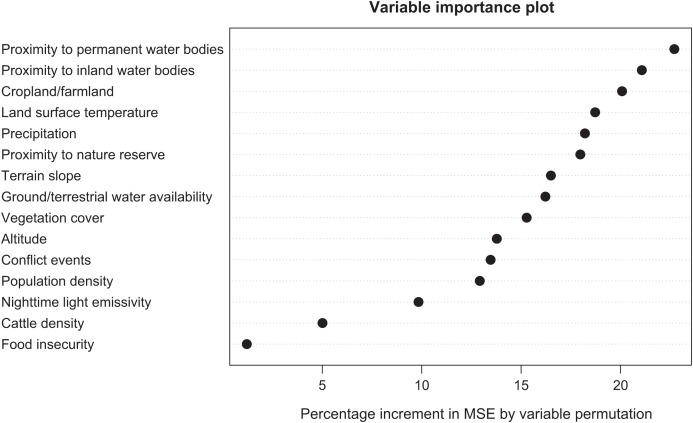
Variable importance as measured by the percentage increment in mean square error (MSE) by variable permutation.

Proximity to permanent and inland water bodies, areas designated as farmland/cropland, land surface temperature, and precipitation estimates were the five most important correlates on Guinea worm infection (Figure 4).

### Marginal effects plots of covariates.

**Figure 5.**
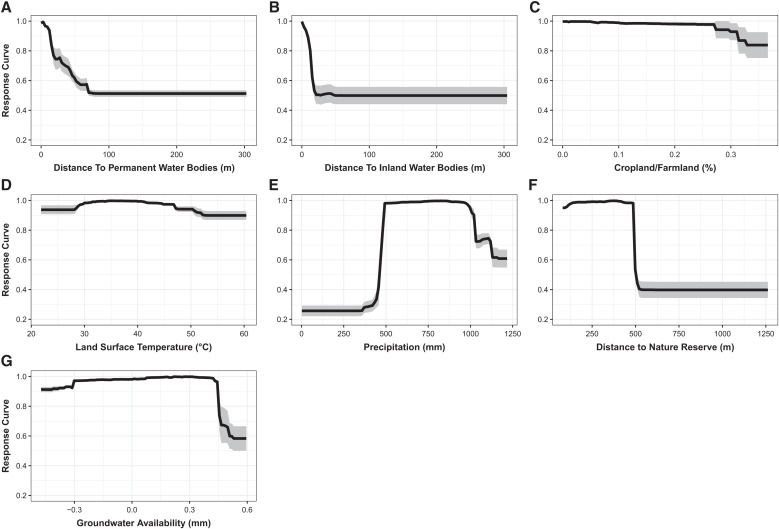
(**A–G**) Marginal effects plots for covariates included in the ensemble. The *y* axis is the response (i.e., probability of detection of Guinea worm infection), and the *x* axis is the full range of covariate values. The black lines represent the mean marginal effects, and the grey shading indicates the 95% bootstrap CIs.

Marginal effects plots indicate that the probability of detection of Guinea worm infection decreases with increasing distance from permanent and inland water bodies and areas designated as nature reserves (Figure 5). These plots also suggest that annual rainfall estimates of between 500 and 1,000 mm are optimum for the detection of Guinea worm infection in endemic communities in the area of Chad under surveillance.

### Risk maps of environmental suitability of Guinea worm disease in Chad.

**Figure 6.**
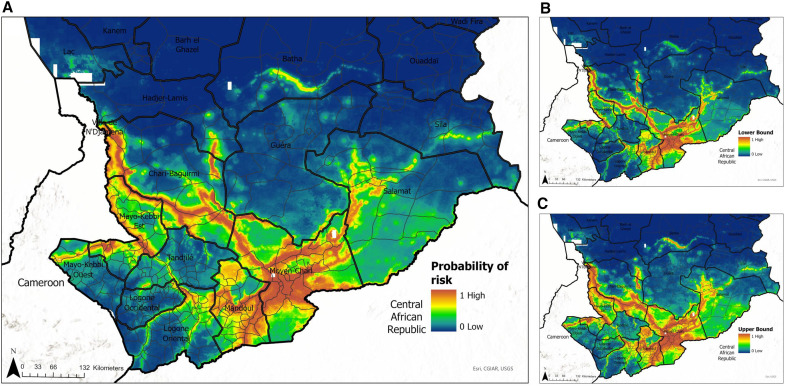
(**A**) Mean predicted probability of Guinea worm infection at 1 km × 1 km resolution. (**B** and **C**) Lower and upper confident limits, respectively.

Predicted risk estimates of Guinea worm infection were projected on a map of the geographical extents of Chad. Risk predictions indicate areas that are environmentally suitable for Guinea worm transmission as explained by the covariates that were included in the model. The probability of Guinea worm infection was higher in the communities within close proximity to the Chari River, in keeping with surveillance data that were used in model development (Figure 6). The model was then used to predict the probability of infection in areas without surveillance data. We also identified potential new areas that share similar ecological characteristics with areas of known infection.

**Figure 7.**
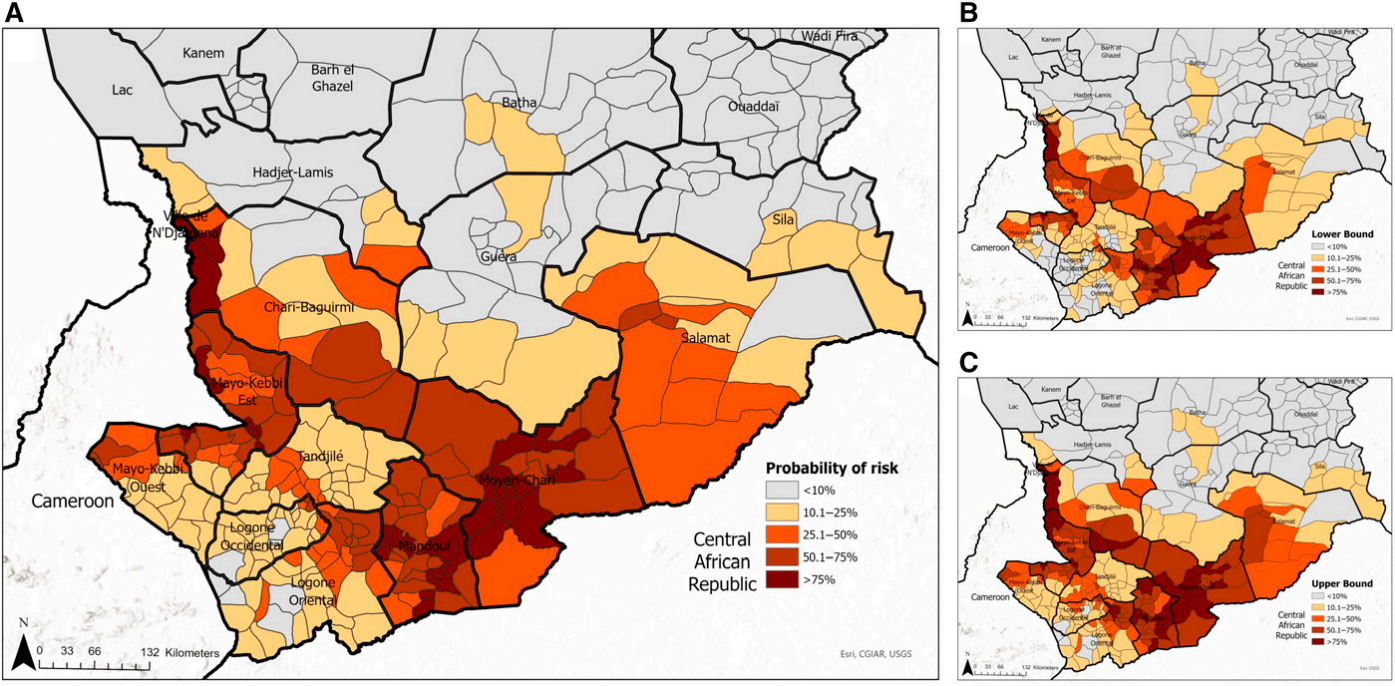
(**A**) Mean predicted probability of Guinea worm infection stratified by district. (**B** and **C**) Lower and upper confident limits, respectively.

The aggregated risk map by sub-prefecture (i.e., district) shows that districts within Moyen-Chari and Mandol regions to the east and Mayo-Kebbi Est and Chari-Baguirmi regions to the west have the highest risk of Guinea worm infection (Figure 7). Also, of note are districts within the Salamat region, particularly in areas bordering Central African Republic.

**Figure 8.**
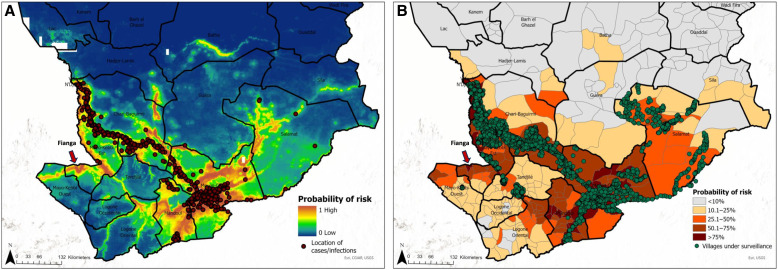
Maps of mean predicted probability of Guinea worm infection. (**A**) Gridded 1 km × 1 km resolution maps overlaid with location of villages reporting Guinea worm infection in all hosts from 2010 to 2022. (**B**) Maps stratified by district overlaid with location of villages under surveillance in 2022. Note the arrow pointing to an area that was predicted as high risk that subsequently began reporting new infections in March 2023.

To highlight the newly identified high-risk areas, the locations of the infection data from 2010 to 2022 used to train the models (Figure 8A) and the villages under surveillance in 2022 have been overlaid onto the map showing the mean predicted probability of infection.

## DISCUSSION

Our model predicts environmental suitability for the detection of Guinea worm infections beyond the areas that have reported Guinea worm infections as well as beyond areas that are currently under active surveillance. Unlike other NTDs, there is a dearth of geospatial prediction maps of Guinea worm. A recent review by Schluth et al.^38^ highlighted the lack of geospatial data for *D. medinensis.*

This work shows that communities at higher risk of Guinea worm infection in Chad are located near the border with Cameroon, close to permanent rivers, inland waterbodies, and nature reserves, as well as areas where the average annual rainfall estimates are between 500 and 1,000 mm. In addition to building pixel-level continuous maps at 1 km^2^ resolution, we aggregated estimates at the district level. Although maps that depict gridded continuous surfaces are important to highlight intra-district heterogeneities in risk estimates, presenting these estimates at the district level might be more useful to guide programmatic decisions, including deployment of relevant intervention measures and resources.

Our maps accurately captured the known spatial distribution of known Guinea worm infections in Chad. Infections are predominantly localized in the southern regions, clustering along the Chari River from the west (near N’Djamena) to the Moyen-Chari region, close to the border with Central Africa Republic. In addition, we have identified several new areas that are classified as being ecologically suitable for Guinea worm transmission. Results from this study grant the CGWEP a unique opportunity to get ahead of Guinea worm surveillance in the final, challenging phases of the campaign. Typically, the geographic expansion of active surveillance has been reactionary in nature, where villages are added to the active surveillance system only after the discovery of new infections in humans or in animals. Here, we identified several new areas where the program might consider preemptively initiating active surveillance, carrying out spot check case/infection sweeps, ramping up health education measures, and/or distributing cloth/pipe filters. These intervention measures when deployed alone or in combination can contribute to faster outbreak response and prevent outbreaks, especially in districts that are only currently being passively monitored for cases or infections.

In order to illustrate the newly predicted high-risk areas, we have plotted location of the infection data used in the modeling and locations of villages under active surveillance in 2022 over the mean predicted probability of infection. Several districts that did not report any cases or infections from 2010 to December 2022 and are not covered under the active surveillance system have been predicted to have ecological characteristics suitable for Guinea worm transmission (see Figure 8B). Of course, Guinea worm transmission is not a spontaneous event; rather, transmission only occurs when an uncontained infection has been introduced or imported into a village. Interestingly, in March 2023, several infections in dogs were reported for the first time in the district of Fianga, along the border with Cameroon. We note that this district did not have observed infection data that was used in the initial model building, but that our models predicted Fianga to be at high risk for Guinea worm transmission. This is a classic example of the usefulness of predictive models, and in some ways, serves as a validation or ground-truthing of our model predictions in that area. At the time of writing (March 2023), necessary intervention measures had been deployed to Fianga as well as neighboring districts on both the Chad and Cameroon sides of the border.

The clustering of infections as indicated by the predicted risk maps presented in this work is corroborated by the semi-variogram analysis that indicated a spatial range of infection of approximately 30 km. A previous spatiotemporal study that analyzed genomic and surveillance data in Chad found that the median range of genetic relatedness between pairs of infections was between 18 and 50 km.^39^ Although these results suggest that Guinea worm infections are rather focal in space, the possibility of importation of cases or infection from areas outside of this range must not be disregarded, as this has been recently observed in an outbreak in Salamat region.^40^ To this end, CGWEP, through a vast network of program staff, conducts narrative case investigations that retrospectively record travel history during the estimated period of infection for every confirmed Guinea worm infection. In future analyses, we aim to explore the potential of including these travel history data or other indicators of migration as a covariate in our models.

Although target product profiles for Guinea worm diagnostic tools are still in draft form, when they are finalized, they will provide recommendations for sensitivity and specificity requirements for reliable diagnostic tools to enable 1) the detection of early or pre-patent infection in animals and 2) the detection of *D. medinensis* in environmental media such as water and/or in aquatic animal transport hosts.^41^^,^^42^ However, field validation is required before widespread usage of any newly identified diagnostic tools. Moreover, knowledge of varying endemicity levels or risk distribution across vast spatial extents is necessary to test diagnostic tools under varying endemicity scenarios. Therefore, maps presented in this work provide a valuable tool for assessing risk levels and may serve as a guide for selection of diagnostic testing sites.

We considered several climate, environmental, and sociodemographic variables as predictors in our model. Proximity to surface water (i.e., permanent and inland water bodies) was the most important variable in the model. Although it is plausible to assume that the route of infection for humans and animals was by drinking water contaminated with infected copepods, it is also possible that proximity to these water bodies may be indicative of fishing villages and communities. This assumption possibly holds true given that infection counts in dogs far surpassed infections in other species that were used to build our model. Also, it is highly likely that domesticated and wild animal hosts acquired infection by eating raw or undercooked fish and fish entrails^6^^,^^7^ due to increased access to these food types in fishing villages. This mode of exposure is suspected to be the most predominant route associated with acquiring Guinea worm infection in areas classified as fishing villages, as corroborated by Richards et al.^43^ This association might have been clearer if we were able to account for villages explicitly devoted to fishing.

We included groundwater-level/terrestrial water as a predictor of Guinea worm infection in our model. This was considered as a proxy for water wells or boreholes around primary residences (i.e., areas with an abundance of groundwater were more likely to have water wells, thus not having to depend on ponds and lakes for their drinking water). This hypothesis is in keeping with Hunter’s study in Ghana.^44^ Perhaps the lack of clear spatial correlation between groundwater-level/terrestrial water and Guinea worm infection here may be another indication that the predominant mode of transmission is associated with eating aquatic animals, not by drinking contaminated water.

A previous case-control study that was conducted in Chad aimed to identify potential human risk factors after the program began reporting cases after a 10-year absence. This study found that secondary water sources were a more significant risk factor than primary water sources.^45^ This may imply that infection was more likely to be acquired outside of areas of primary residence, such as in farmland or crop fields where access to safe water is limited and people and animals are more likely to use unsafe surface water sources. Perhaps, this explains why the covariate used to classify areas used for farming activities was an important predictor of Guinea worm infection in our model. It is worth noting that most inhabitants of Guinea worm–endemic villages are farmers (either for subsistence or commercial purposes)^27^; therefore, it is thought that infection was also more likely to be acquired in crop fields because of the unavailability of clean drinking water sources in these fields.

Guinea worm infection follows a clear seasonal pattern in Chad, with peak transmission occurring in the rainy season from the months of April to July. This is indicative of the prominence of our rainfall covariate, which emerged as an important correlate of infection. Adequate rainfall will translate to the formation of more temporary water ponds, which may serve as seasonal water sources within the villages; however, annual rainfall estimates >1,000 mm may result in flooding, thus washing off breeding sites of copepods. Moreover, it is critical to note that the seasonal peak of Guinea worm cases and infection also corresponds with mass fishing events along the Chari River during the rainy season.

Furthermore, we considered land surface temperature in our model as opposed to air temperature. Although air temperature may also highlight seasonal trends in transmission to some extent, studies have shown that the influence of land surface temperature on the ecology, survival, and fecundity of copepod populations is significant, especially on the ability to transmit Guinea worm infection. Food insecurity and conflict events were not found to be important correlates of Guinea worm infection in Chad. It will be interesting to assess the effects of these covariates in other Guinea worm endemic countries such as Mali and South Sudan, which have had more frequent occurrences of conflict events, civil unrest, internally displaced populations, and disruption in commodity supplies, often resulting in acute food shortages.

We acknowledge some caveats and limitations in this work. First and foremost, the prediction of environmental suitability alone does not guarantee the occurrence of Guinea worm transmission. Also, there are other factors, such as individual and community-level cultural and behavioral factors, that species distribution models do not capture. These factors might help identify geographical areas with suitable environmental conditions that may be at even greater risk for the occurrence of Guinea worm infection. In addition, the dependent variable that was used to build the model was village-level presence of Guinea worm infection derived from surveillance data. Therefore, the reliability of this dependent variable rests upon the sensitivity of the surveillance system. Although we are confident in the system’s ability to detect human cases,^46^ detecting canine cases is more challenging,^47^ especially in insecure areas, villages close to country borders, and within nomadic populations. Finally, we have not accounted for intervention in our models. The purpose of this work was to demonstrate the overall distribution of Guinea worm infection in Chad, but in future work we intend to evaluate the effectiveness of intervention and build temporal maps that describe the change in infection distribution over many years.

## CONCLUSION

Overall, this work presents a unique resource and provides the most comprehensive maps for Guinea worm distribution and for prediction of environmental suitability in Chad. Improved knowledge of the distribution of infections is useful for the extension of surveillance and other necessary intervention measures to newly identified areas and intensified intervention efforts in previously known endemic areas. As the CGWEP moves toward elimination, the methods and results in this study will inform surveillance activities and help optimize the allocation of intervention resources.
